# An overview of fasciolosis in human and cattle populations in New Valley, Egypt

**DOI:** 10.3389/fvets.2025.1572946

**Published:** 2025-08-20

**Authors:** Abeer A. Khedr, Sara Salah Abdel-Hakeem, Wafaa G. Mahmoud, Sally Salah Abdel-Hakeem, Ahmed M. Al-Hakami, Mohammed E. M. Tolba, Salwa Mahmoud Abd-Elrahman, Mervat M. Khalifa

**Affiliations:** ^1^Department of Parasitology, Faculty of Veterinary Medicine, New Valley University, El-Khargah, Egypt; ^2^Department of Biosciences, Durham University, Durham, United Kingdom; ^3^Parasitology Laboratory, Zoology and Entomology Department, Faculty of Science, Assiut University, Asyut, Egypt; ^4^Department of Pathology, South Egypt Cancer Institute, Assiut University, Asyut, Egypt; ^5^Department of Clinical Microbiology and Parasitology, College of Medicine, King Khalid University, Abha, Saudi Arabia; ^6^Department of Parasitology, Faculty of Veterinary Medicine, Assiut University, Asyut, Egypt; ^7^Department of Medical Parasitology Faculty of Medicine, Assiut University, Asyut, Egypt

**Keywords:** *Fasciola hepatica*, retrospective, human, demographic, histopathology, genetic characterization

## Abstract

**Introduction:**

Fascioliasis, a significant global zoonotic disease caused by trematode parasites of the genus *Fasciola*, affects various livestock species.

**Aim:**

This study aimed to identify demographic, epidemiological, clinical manifestations, pathological, and genetic characteristics in New Valley, Egypt's human, and cattle populations.

**Methods:**

This study is made of two parts, the first part is a cohort study of 1000 cattle slaughtered at three abattoirs in El Kharja, El Dakhilah, and El Farafra from February 2023 to January 2024. A retrospective analysis of patients visiting El Kharja hospital with clinical symptoms and confirmed with coprological and radiological examinations.

**Results:**

The study revealed a high prevalence in cattle (23%) and humans (3.6%). Enrolled human fascioliasis was diagnosed in 58.3% and 41.7% by coprological and radiological analysis, respectively. A 66.7% of enrolled cases were females, with a median age range of 37 ± 13 years old. Treatment outcomes demonstrated a response of 25%, 50%, and 25% to single, two, and three doses of the treatment, respectively. In cattle, infection rate was 20.0% in females compared to 24.9% in males with low prevalence in animals <1 year (12.7%) and high in animals >3 years (30.4%). Autumn had the highest prevalence (28.7%), whereas summer had the lowest prevalence (18.0%). A significant difference in the prevalence of fasciolasis was observed between human and animals. Morphological and histopathological analysis elucidated acute and chronic manifestations of hepatic fascioliasis with ectopic migration to cattle lung tissue. Genetic characterization of ectopic worm confirmed *Fasciola hepatica* infection, with genetic similarity to human isolates from Iran.

**Discussion:**

The study highlights the importance of one health approach in understanding and managing fascioliasis.

## Introduction

Fascioliasis, a global food-borne zoonotic affliction caused by trematode parasites of the genus *Fasciola*, significantly impacts livestock, including humans and animals (1). The World Health Organization (WHO) has classified fasciolosis as a neglected tropical disease, and it is the most geographically distributed parasitic infection (2). Simultaneously, it emerges as a substantial zoonotic disease in South America, Egypt, Iran, and Vietnam, with reports estimating global infections (3). Occurrence of fascioliasis is closely linked to freshwater mollusks of the genus *Lymnaea* spp., which serve as intermediate hosts for the liver fluke (4). Therefore, infection is acquired when animals ingest forage or water contaminated with *Fasciola* spp. metacercariae, the infective larval stage of the parasites (5). Humans can be infected by ingestion of contaminated vegetables, water, and occasionally through consumption of undercooked or raw liver products (6–8). Fascioliasis prevalence is influenced by a range of ecological factors, including the presence of the intermediate host *Lymnaeidae* snails; consequently, the prevalence of the disease exhibits significant variability worldwide (9). In both human and animal infections, the clinical signs of fasciolosis depend on the period of infection (either invasive/acute or chronic) and are related to the level of damage to the liver (3).

*Fasciola* infection leads to hepatic lesions, fibrosis, and chronic bile duct inflammation with many subclinical infections remaining (10). Ectopic or aberrant fasciolosis has been shown to occur when migrating immature flukes find their way to other organs, most commonly elsewhere in the gastrointestinal tract but also the abdominal wall, heart, lungs, and occasionally, the eyes or brain (11). The economic impact of *F. hepatica* infection in cattle and sheep is substantial, estimated at over 3 billion USD annually on a global scale including Egypt (6, 12). The economic losses were categorized as direct losses, which consist of drug costs, labor, and liver condemnation at abattoirs, and indirect losses associated with decreased productivity, such as reduced production, feed conversion efficiency, weight gain, and fertility (13).

Diagnosing *Fasciola* infection requires a multifaceted approach, incorporating various methods. These include coprological examination to identify *Fasciola* eggs in stool samples, alongside imaging techniques like abdominal ultrasonography or computed tomography (14). In animals, visceral inspection may also be employed, where the presence of worms in the liver serves as a diagnostic indicator (15, 16). Considering the complexity of *Fasciola* characterization through morphological examination, molecular approaches have been used to identify this parasite with higher accuracy (17).

The Internal transcribed spacer 1 *(ITS1)* region of the nuclear rDNA cluster in *Fasciola* species is a valuable tool for studying their molecular biology and evolutionary relationships (18). This region, which can be amplified and sequenced using universal primers, provides a high level of variation, making it useful for phylogenetic studies (19). Additionally, the *ITS* region has been used to genetically characterize *Fasciola* species, distinguishing between *Fasciola hepatica* and *Fasciola gigantica* (20). There are few studies on human and animal fascioliasis in the New Valley Governorate, Egypt. Recently, Hassan et al. (21) reported that the prevalence of fascioliasis was 0% in humans and 1.6% in animals based on coprological examinations. Moreover, sheep had a higher infection rate of 2.9% compared to cattle 1.3% with no significant association between infection and factors such as species, age, sex, locality, or time of infection (21). Moreover, Elshraway et al. (22) reported high prevalence rate of fasciolasis (30.88%) in cattle slaughtered in El-Kharga oasis, the New Valley Governorate, Egypt. The study aims to evaluate the prevalence rates and demographic profiles of humans and cattle affected by fascioliasis in New Valley Governorate, Egypt. Furthermore, evaluate the genetic similarities between human and cattle parasites. Data were collected over a 1-year period from infected cattle and human patients who had clinical symptoms and treatment outcomes. This research endeavors to contribute to the understanding of fascioliasis transmission dynamics, informs public health strategies by highlighting affected demographics, offers insights into disease manifestations from necropsy results, and inform public health interventions for effective control and prevention of the disease in the study area and beyond. On the other hand, establish connections between human and cattle infections, indicating zoonotic transmission.

## Materials and methods

### Ethical approval

Animal data and sampling were conducted according to the guidelines of the OIE standards and university guidelines of the Committee of the Faculty of Veterinary Medicine, Assiut University, Egypt (approved number: 06/2024/0172). The study was conducted following the ARRIVE (Animals in Research: Reporting In Vivo Experiments) criteria (23). Retrospective patient data were guaranteed to remain confidential, while data sheets were coded with numbers to maintain anonymity and were conducted in accordance with the ethical guidelines of the Helsinki Declaration by the Medical New Valley Ethics Committie (Approval No. 20241230013), Faculty of Medicine, New Valley University, Egypt.

### Study area

**Figure 1:**
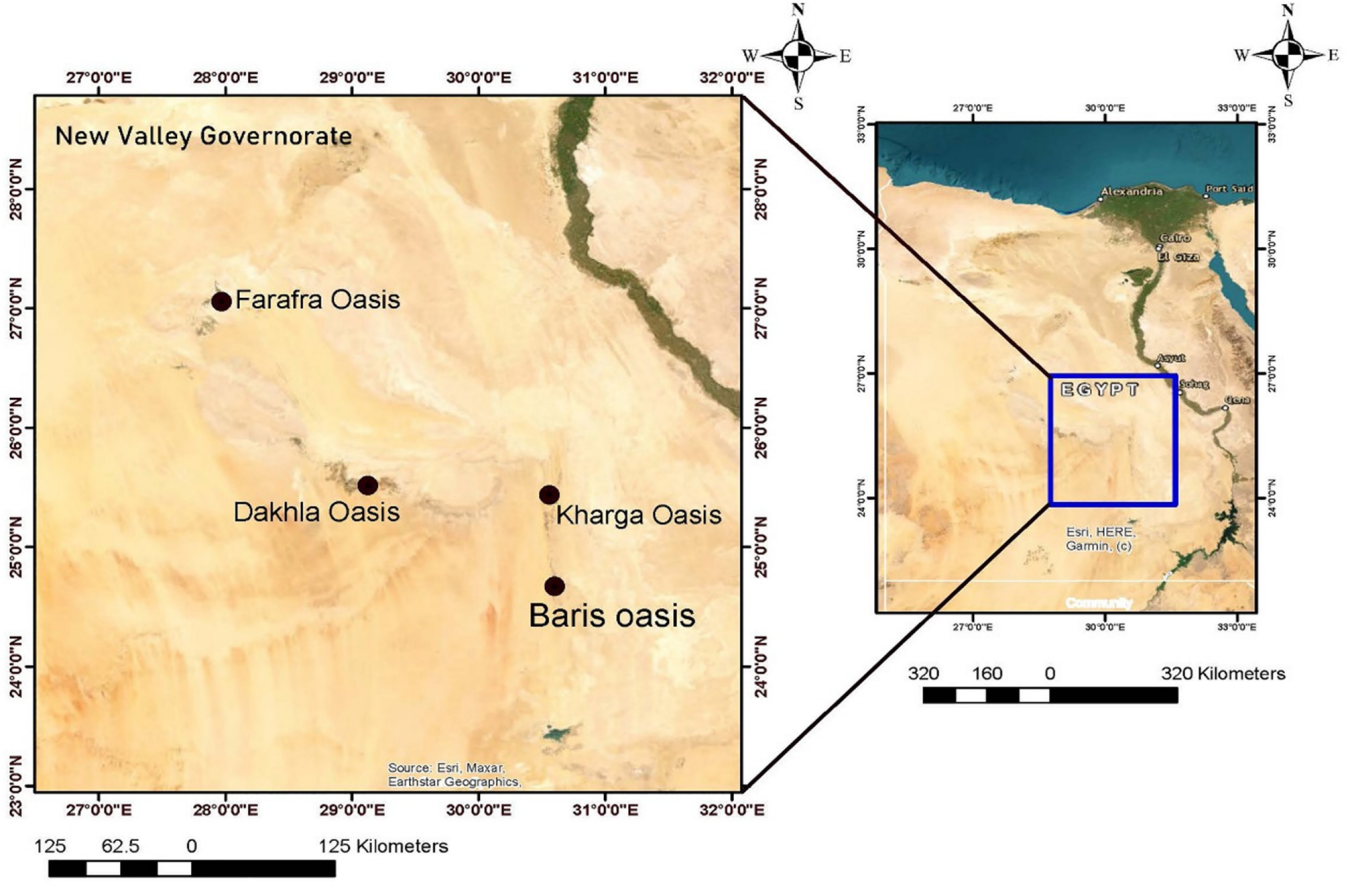
Map of Egypt highlighting New Valley Governorate with a detailed view of four oases: EL-Farafra, EL-Dakhla, EL-Kharga, and Baris. The inset shows the location within Egypt, showcasing major cities and geographic features. Compass rose and scale bars are included.

The New Valley Governorate is located in southwestern Egypt at approximately 24°2′45.70“N latitude and 27°9′44.91”E longitude (Figure 1). It has an arid climate and extensive desert and oasis terrain. It is the largest area in Egypt (440.098 km^2^) with the lowest population density (2 people per km^2^) and a poverty rate of 52.6%. It includes five cities: El Kharga, El Dakhla, Baris, Abo Monqar, Gharb Elmohoub, and El Farafra Oasis.

### Data collection

Across sectional study consisted of two parts, the first part is a cohort study of cattle slaughtered at three abattoirs from February 2023 to January 2024. A total of 1,000 cattle were examined for fascioliasis, including 602, 222, and 176 in El Kharja, El Farafra, and El Dakhilah, respectively. The enrolled animals comprised 610 males and 390 females, spanning different age ranges including calves (>1 year), young (1–3 years), and adults (>3 years). Records of all fasciolosis cases that have been identified and confirmed (230 cases) through necropsy findings, meat inspection, and exclusion of cattle liver. Demographic data regarding sex, age range, and season of collection (Summer from June to August, Autumn from September to November, Winter from December to February, and Spring from March to May) (24) were considered. Necropsy records of concurrent infections, histopathology, parasitological findings, ectopic migration, and molecular identification of ectopic worms were assessed.

In the second part, based on our results of animal infection, we performed a retrospective cohort study on 334 patients visiting El Kharja Hospital with symptoms such as abdominal pain, jaundice, and fever. Records of coprological and radiological examinations were examined for final diagnosis. Demographic data were collected based on sex, age range, drug response, and follow-up coprological and radiological examination. The drug efficiency was evaluated based on routine diagnosis, egg clearance, according to the Ministry of health guidelines.

### Postmortem examination and gross examination

The postmortem examination protocol began with a visual assessment of the outer carcass surface to identify any abnormalities. Subsequently, the abdominal cavity was opened to conduct a comprehensive inspection of internal organs, particularly focusing on the liver, lungs, and intestine. During the examination, a non-toothed forceps was used to collect any detected parasites. All inspection visits to the slaughterhouse were conducted under the supervision of a licensed veterinarian to ensure precise observations. Following the examination, the samples were collected, carefully stored, and transported to the Laboratory of Parasitology, Faculty of Veterinary Medicine, New Valley University, Egypt for further detailed analysis.

### Morphological analysis

A morphological analysis of the liver flukes was conducted using a light microscope. The specimens were stored in a refrigerator at 4°C until they reached a state of complete relaxation. Then, the flukes were fixed in 70% ethanol and stained in acetic acid alum carmine (25). The flukes were identified according to the key by Gibbons et al. (26).

### Histopathological examination

The liver and lung specimens of the infected animals underwent histopathological analysis according to Abdel-Hakeem et al. (27). The specimens were fixed in 10% neutral buffered formalin, dehydrated in an ascending concentration of ethyl alcohol, and cleared in xylene for 24 h. The specimens were embedded in paraffin and sliced into 4–5 μm sections using a rotatory microtome. The sections were meticulously placed on glass slides and subjected to hematoxylin and eosin staining. The examination was conducted using a light microscope (Olympus BX43F, Tokyo 163-0914, Japan), and the captured images were obtained through a camera (Olympus, EP50, Tokyo, Japan) at the Photomicrograph Lab of the Department of Parasitology, Faculty of Veterinary Medicine, Assiut University (28).

### DNA extraction and PCR amplification

Genomic DNA was extracted from immature flukes present in the lung tissue using QIAamp DNA Mini Kits (Qiagen, USA) as per the manufacturer's guidelines. The *ITS1* fragment was amplified through PCR (29), employing the forward primer 5′-TTGCGCTGATTACGTCCCTG-3′ and reverse primer 5′-TTGGCTGCGCTCTTCATCGAC-3′. The PCR Master Mix was prepared with the Emerald Amp GT PCR Master Mix (Takara) Code No. RR310A following the manufacturer's instructions. The reaction was composed of 12.5 μl of the 2x premix, 5.5 μl of PCR-grade water, 1 μl each of the forward and reverse primers (20 pmol each), and 5 μl of template DNA (measured at 50 ng/μL), resulting in a total reaction volume of 25 μl. For amplifying the target gene, the temperature and time parameters comprised an initial denaturation at 95°C for 5 min, followed by secondary denaturation at 94°C for 30 s, annealing at 53°C for 40 s, and extension at 72°C for 45 s. This cycling process was repeated for 35 cycles, with a final extension step at 72°C for 10 min. Following the preparation of the reaction mixture with a total volume of 25 μl, each sample underwent separation on a 1.5% agarose gel using electrophoresis. The PCR products were visualized and captured using a UV radiation detector to confirm successful amplification (30).

### Purification of PCR products

The PCR products were purified using the QIAquick PCR Product Extraction Kit (Qiagen Inc., Valencia CA). The purification involved the PCR sample and Buffer BP1 with a volume of 1:5, applying the mixture to a QIAquick spin column, washing with buffer PE, and eluting the purified DNA using Buffer EB or water. This purification process typically took around 10 min and resulted in the production of purified PCR products suitable for subsequent applications (25).

### Sequence and phylogenetic analysis

The positive PCR clones were selected for sequencing. The purified PCR products were subjected to sequencing in both the forward and reverse directions using an automated DNA sequencer from Applied Biosystems (ABI, 3130, USA). The sequencing reactions were performed using the BigDye Terminator v3.1 Cycle Sequencing Kit from PerkinElmer/Applied Biosystems, Foster City, CA. Upon successful sequencing, the obtained sequences were compared to existing sequences available in GenBank using the NCBI BLAST program. Phylogenetic analysis was performed using the CLUSTAL W multiple sequence alignment program (version 2.1 of the MegAlign module) (31). The evolutionary relationship among *ITS1*-rDNA genes was further elucidated by constructing phylogenetic trees employing both the maximum likelihood (ML) method and the neighbor-joining distance method in MEGA11, with a confidence level of 1,000 bootstrap replicates (32). Finally, the evolutionary relationships and genetic similarities/differences between our sample and various populations were visualized using Interactive Tree of Life (ITOL) v6.6 (33).

### Data analysis

The gathered data was meticulously entered into a spreadsheet in Microsoft Excel 2010 and then analyzed using Statistical Package for the Social Sciences (SPSS, version 20) for Windows 10 (34). Descriptive statistics, frequencies, and percentages for categorial variables were used to determine the prevalence of *Fasciola hepatica* infection. Prevalence rates and 95% confidence intervals (CIs) were calculated using the Wilson score method. Chi-square tests were performed to determine associations between infection rates in each variable (gender, age, season, and abattoir). Univariate binary logistic analysis was conducted to estimate the odds ratios (OR) with 95% confidence intervals (CI) with infection as the binary outcome. Reference groups were chosen based on the lowest infection rates and used to estimated odds ratios for each variable. A multivariate binary logistic regression analysis was performed using backward step-wise selection method, starting with all variables (gender, age, season, and abattoir). The nonsignificant variables (*P* > 0.05) were excluded, and only significant variables (*P* < 0.05) remained. The same reference groups as in the univariate analysis were maintained. Model fit was evaluated using the Hosmer-Lemeshow goodness-of-fit Test and Negelkerke *R*^2^. *P*-value of less than 0.05 was indicative of statistical significance.

## Results

### Data of enrolled humans and cattle

As shown in Table 1, a total of 12 (3.6%) human patients were positively diagnosed to fascioliasis and included in the study, with 7 (58.3%) diagnosed through coprological analysis, whereas radiological diagnosis was done in 6 (41.7%). The enrolled cases showed significant differences between the infection and gender. They included 8 females (66.7%) and 4 (33.3%) males with a median age of 37 ± 13 years and a range between 15 and 65 years. According to the protocol of the Egyptian Ministry of Health and Population, a single dose of triclabendazole (10 mg/kg) was used in the treatment of human fascioliasis. In this study, the response was reported in 3 patients (25%) to a single dose, 6 patients (50%) to two doses, and 3 (25%) to three doses of the treatment (Table 1).

**Table 1:** Demographic data of human patients diagnosed with fasciolasis.

**Variables**	**No. 12**	**%**	**Chi-square (χ^2^)**	***P-*value**
**Gender**				
Male	4	33.3%	12.190(df = 1)	0.001
Female	8	66.7%		
**Age**				
15–30 years	3	25%	0.163(df = 2)	0.922
31–42 years	5	41.7%		
43–65 years	4	33.3%		
**Drug response**				
One dose	3	25%	2.788(df = 2)	0.248
Two doses	6	50%		
Three doses	3	25%		
**Diagnosis**				
Coprological	7	58.3%	6.370(df = 1)	0.012
Radiological	5	41.7%		

A total of 1,000 cattle were examined for fascioliasis across three abattoirs (El-Khargah, El-Dakhla, El-Farfra) in Egypt were included in this study. Of these, 610 (61%) were male, and 390 (39%) were female. Age distribution included 229 (22.9%) animals < 1 year, 524 (52.4%) aged 1–3 years, and 247 (24.7%) >3 years. Samples were collected across seasons: autumn (230, 23%), winter (226, 22.6%), spring (227, 22.7%), and summer (317, 31.7%). By abattoir, 602 (60.2%) animals were from El-Khargah, 176 (17.6%) from El-Dakhla, and 222 (22.2%) from El-Farfra. Fecal samples were examined using copro-microscopy to detect Fasciola spp. eggs. Necropsy examination revealed acute fascioliasis in 160 (69.6%) cases, chronic fascioliasis in 70 (30.4%) cases, and ectopic lung migration in 8 (3.5%) cases. Additionally, concurrent parasite infection was observed, with 24 (10.4%) cattle infected with *Paramphistomum* spp., 125 (45.3%) with *Moniezia* spp., and 9 (3.9%) with *Avitellina* spp. Furthermore, parasitic nodules were found due to *Oesophagostomum* spp. infection in 72 (31.3%) cattle.

**Figure 2:**
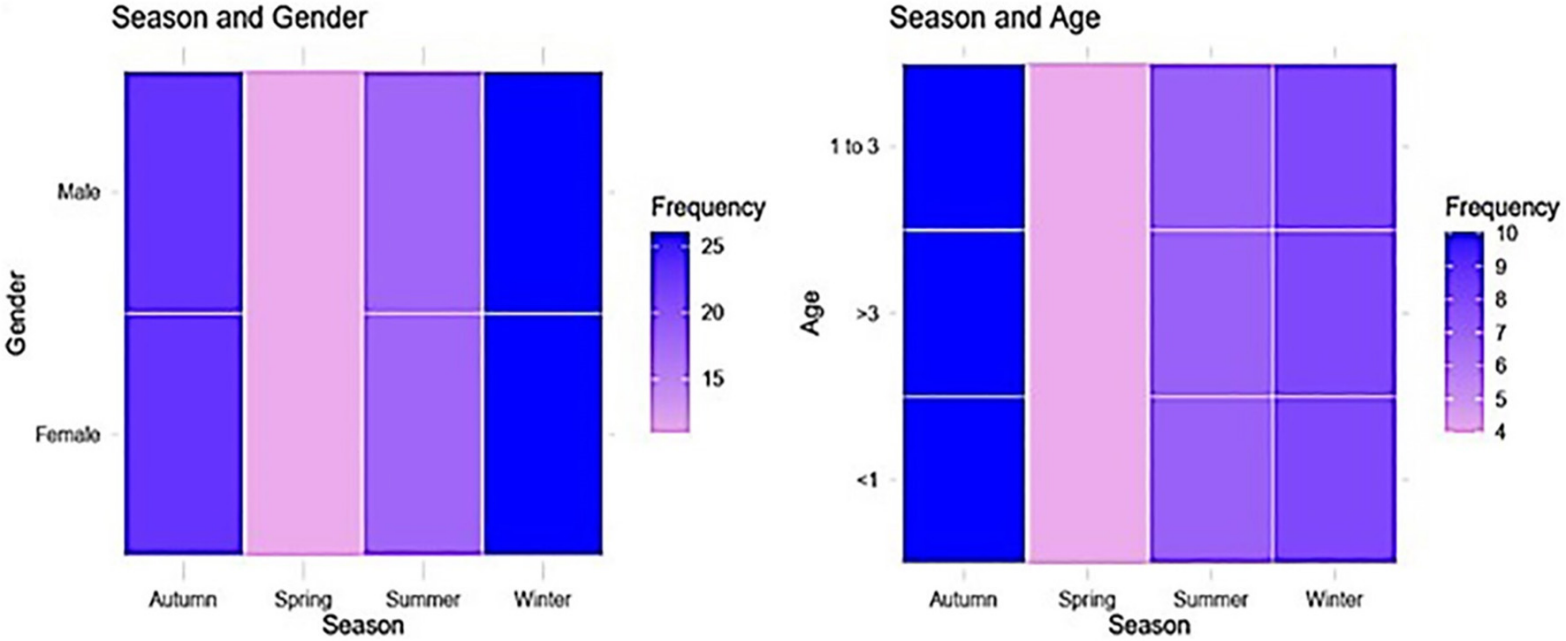
A heat map indicates significant associations between gender and age categories, in relation to season.

Overall, 230 were confirmed with *F. hepatica* infection, with a prevalence of 23% (95% CI: 20.5–25.7%). As shown in Table 2 and Figure 2, infection rate varied by gender, age, season, and abattoir. Among females, 78 of 390 were infected (20.0%, 95% CI: 16.3–24.3%), compared to 152 of 610 males (24.9%, 95% CI: 21.7–28.4%). By age, prevalence was lowest in animals < 1 year (12.7%, 95% CI: 8.9–17.7%, 29/229), followed by 1–3 years (24.0%, 95% CI: 20.6–27.9%, 126/524), and highest in animals >3 years (30.4%, 95% CI: 24.9–36.5%, 75/247). Seasonally, autumn had the highest prevalence (28.7%, 95% CI: 23.2–34.9%, 66/230), followed by winter (24.8%, 95% CI: 19.6–30.8%, 56/226), spring (22.5%, 95% CI: 17.6–28.3%, 51/227), and summer (18.0%, 95% CI: 14.2–22.5%, 57/317). By abattoir, El-Khargah had the highest prevalence (24.8%, 95% CI: 21.6–28.3%, 149/602), followed by El-Farfra (26.1%, 95% CI: 20.8–32.3%, 58/222), and El-Dakhla (13.1%, 95% CI: 8.9–18.7%, 23/176).

**Table 2:** The univariable analysis of the *Fasciola hepatica* infection in the cattle (*n* = 1,000) sampled from 3 different abattoirs of New Valley, Egypt.

**Variable**	**Category**	**Prevalence (%)**	**OR (95% CI)**	**Wald *P* value**	**Correlation**
Gender	Female	20.0%	Ref^*^	–	0.71
	Male	24.9%	1.328 (0.96–1.831)	0.086	
Age	< 1 year	12.7%	Ref^*^	–	0.000
	1 to 3 year	24.0%	2.172 (1.372–3.436)	0.001	
	>3 year	30.4%	2.977 (1.822–4.863)	< 0.001	
Season	Summer	18 %	Ref^*^	–	0.027
	Spring	22.5%	1.323 (0.862–2.033)	0.199	
	Autumn	28.7%	1.834 (1.195–2.815)	0.024	
	Winter	24.8%	1.503 (0.974–2.319)	0.064	
Abattoir	El-Dakhla	13.1%	Ref^*^	–	0.002
	El-Khargah	24.8%	2.187 (1.341–3.566)	0.002	
	El-Farfra	26.1%	2.353 (1.385–3.998)	0.002	

OR, odds ratio; CI, Confidence Interval. Statistically significance P-value < 0.05. Correlation P-value derived from Chi-square tests. ^*^ is the reference group.

Univariate analysis using Chi-square tests identified significant associations between *Fasciola hepatica* infection and age (*P* < 0.001), season (*P* = 0.027), and abattoir (*P* = 0.002), with a marginal association for gender (*P* = 0.071) (Table 2). The univariate logistic regression results (Table 2) showed that animals aged 1–3 years had 2.17 times higher odds of infection compared to those < 1 year (95% CI: 1.37–3.44, *P* = 0.001), and those >3 years had 2.98 times higher odds (95% CI: 1.82–4.86, *P* < 0.001). However, Autumn season had 1.83 times higher odds of infection compared to Summer (95% CI: 1.20–2.82, *P* = 0.024), whereas Spring (OR = 1.32, 95% CI: 0.86–2.03, *P* = 0.199) and Winter (OR = 1.50, 95% CI: 0.97–2.32, *P* = 0.064) showed no significant associations. Compared to El-Dakhla, animals at El-Khargah had 2.19 times higher odds of infection (95% CI: 1.34–3.57, *P* = 0.002), and those at El-Farfra had 2.35 times higher odds (95% CI: 1.39–4.00, *P* = 0.002). Gender and Season showed no significant associations in univariate analysis (*P* > 0.05).

In the multivariable logistic regression analysis (Table 3), a binary logistic regression analysis was performed with the infection status as the dependent to gender, age, season, and abattoir as predicted variables. The final model indicated age and abattoir, as significant variables. Animals aged 1–3 years had 2.10 times higher odds of infection compared to those < 1 year (95% CI: 1.32–3.36, *P* = 0.002), and those >3 years had 2.80 times higher odds (95% CI: 1.70–4.63, *P* < 0.001). Compared to El-Dakhla, animals at El-Khargah had 2.07 times higher odds (95% CI: 1.26–3.41, *P* = 0.004), and those at El-Farfra had 2.17 times higher odds (95% CI: 1.27–3.73, *P* = 0.005). However, gender and season were excluded due to nonsignificant contributions in the multivariate model. The final model fit using the Hosmer-Lemeshow goodness-of-fit Test showed a chi-square value of 7.82 (*P* = 0.451), indicating a good fit. Furthermore, the Negelkerke *R*^2^ was 0.126, suggesting that the model explains 12.6% of the variance in infection status.

**Table 3:** Binary logistic regression of the *Fasciola hepatica* infection in the cattle (*n* = 1,000) sampled from 3 different abattoirs of New Valley, Egypt.

**Variable**	**Comparison**	**OR (95% CI)**	**Wald *P* value**
**Age**			
< 1 year	Ref^*^	1.00	-
1 to 3 year	1–3 years vs. < 1 year	2.103 (1.317–3.360)	0.002
>3 year	>3 years vs. < 1 year	2.803 (1.697–4.630)	< 0.001
**Abattoir**			
El-Dakhla	Ref^*^	1.00	–
El-Khargah	El-Khargah vs. El-Dakhla	2.073 (1.260–3.412)	0.004
El-Farfra	El-Farfra vs. El-Dakhla	2.174 (1.268–3.727)	0.005

OR, odds ratio; CI, Confidence Interval. Statistically significance P-value < 0.05. Model fit indicators: Hosmer-Leme show test: χ^2^ = 7.82, df = 8, P= 0.451; Nagelkerke R^2^ = 0.126. ^*^ is the reference group.

A significant difference (*P* < 0.001) in the prevalence of fasciolasis was observed between human and animals (Table 4).

**Table 4:** Comparison of infection rates between humans and cattle population.

**Species**	**Infected (%)**	**Odds ratio (95% CI)**	**χ^2^**	***P*-value**
Humans	12 (5.2)	Ref^*^ (1.00)	37.67	< 0.001
Cattle	230 (23)	5.45 (2.99 – 9.93)		

X^2^, Chi-square. Statistically significance P-value < 0.05. ^*^ is the reference group.

### Morphometric analysis of the retrieved specimens

**Figure 3:**
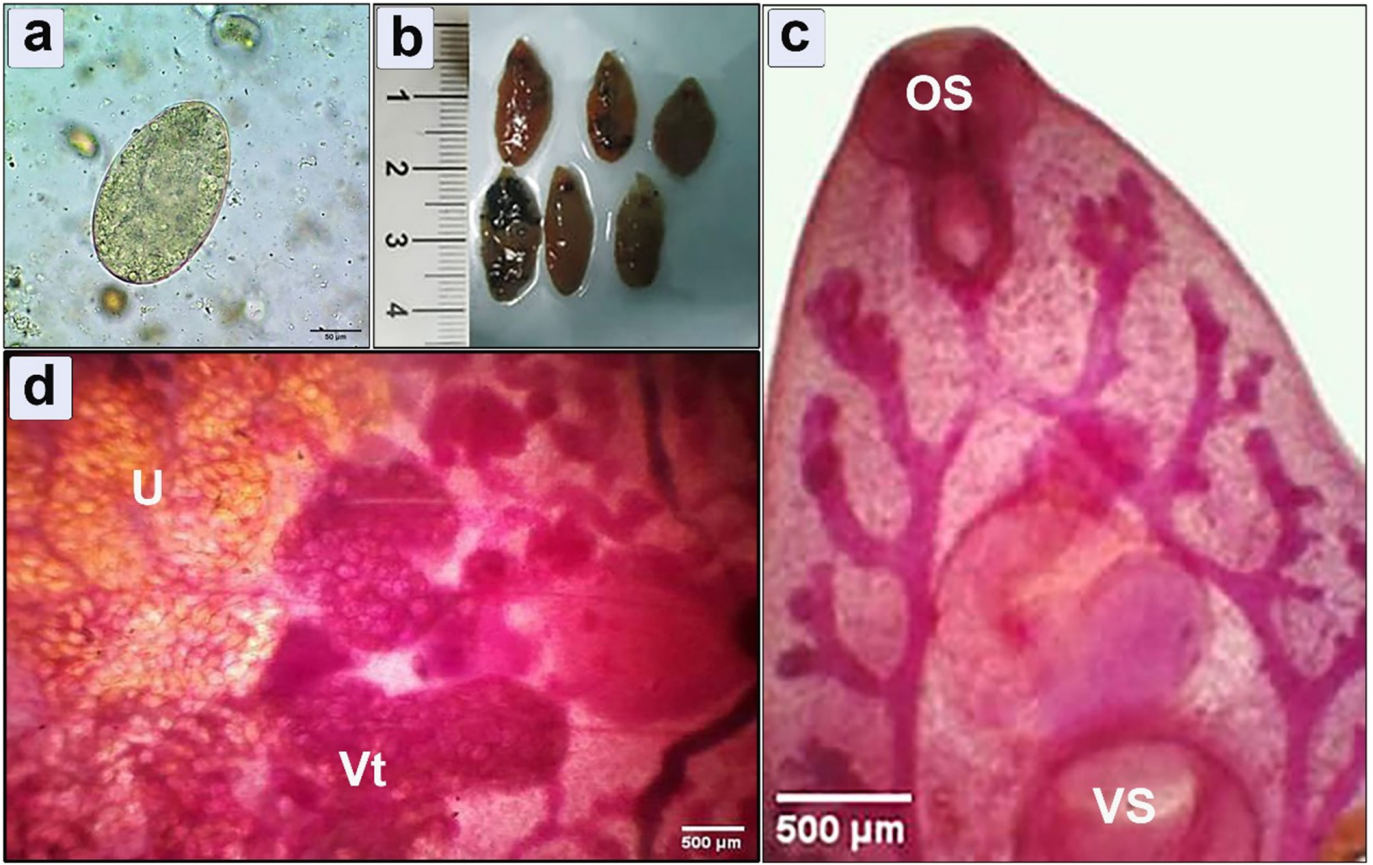
Morphometric examination of the collected specimens showing **(a)** *Fasciola* spp. egg retrieved from human infected feces (bar = 50 μm); **(b)** Macroscopic examination of flukes isolated from hepatic parenchyma of the infected cattle; **(c, d)** Microscopic examination shows cephalic cone, oral sucker (OS), ventral sucker (VS), uterus (U), and vitelline gland (Vt) (bar = 500 μm).

Coprological examinations of human patients exhibited the diagnostic operculated, yellowish-colored egg of fasciola (Figure 3a). In the postmortem examination of cattle, the retrieved *Fasciola* flukes exhibited an elongated body with a total length 20 mm ± 5, a distinct reddish-brown color, presenting a smooth and shiny surface (Figure 3b). The microscopic examination showed a prominent cephalic cone with a conspicuous oral sucker (Figure 3c), complemented with a larger ventral sucker positioned toward the middle, uterus, and vitelline gland (Figure 3d).

### Pathological lesions

#### Liver

**Figure 4:**
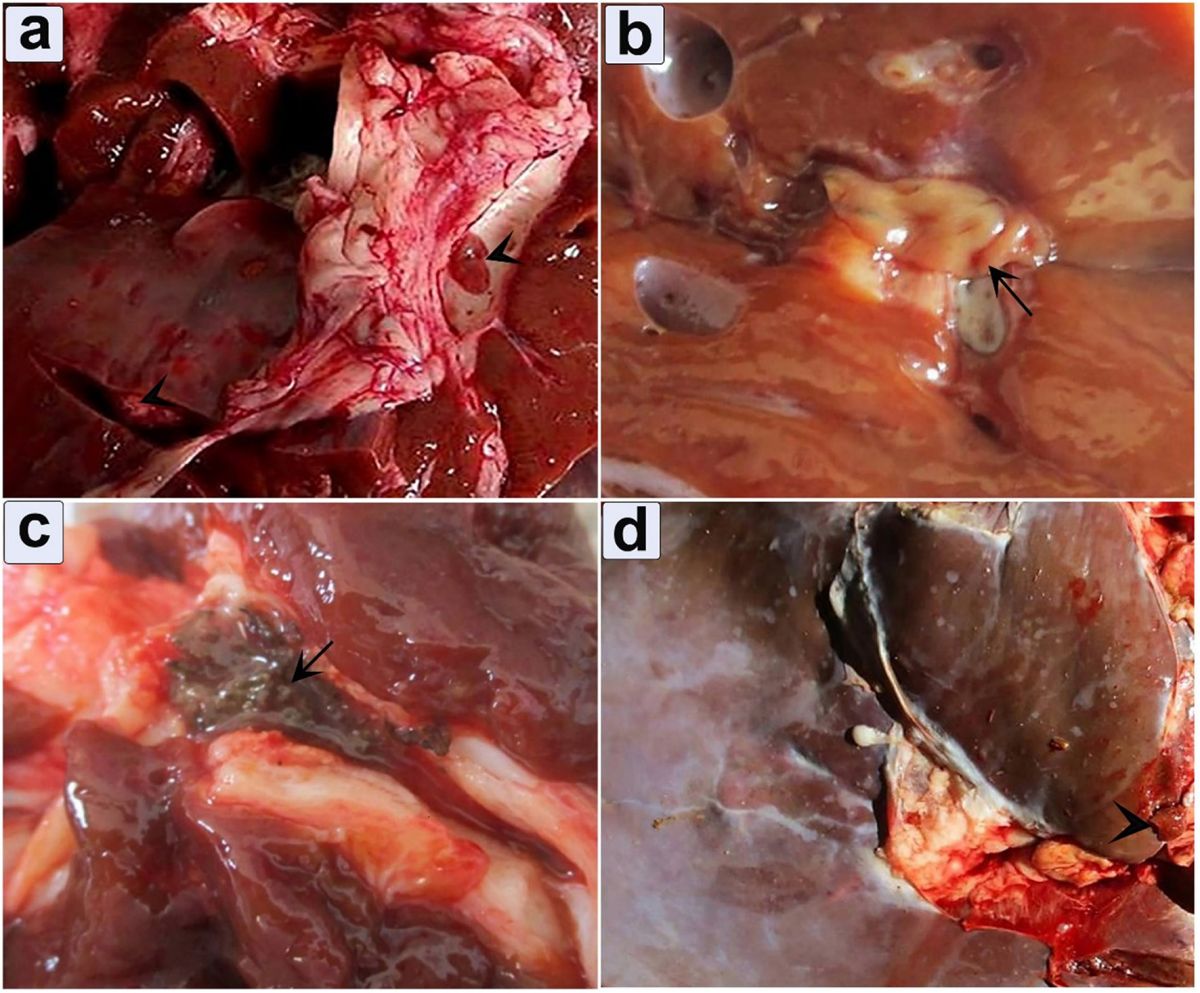
Gross appearance of infected liver illustrates the pathological impact of fascioliasis. **(a)** Fasciola worms within the bile duct, accompanied by extensive bleeding from the cut surface (arrowhead); **(b)** An infected liver exhibits an adult *Fasciola* species adult worm (arrow); **(c)** Hematoporphyrin pigment was observed in the bile duct (arrow); **(d)** Depicting engorgement of the bile duct.

Grossly, two distinct manifestations of hepatic fascioliasis, acute and chronic, were observed (Figure 4). Macroscopic analysis of acute fascioliasis revealed notable features such as enlargement, firmness, congestion, and spontaneous bleeding from the incised surface (Figures 4a, b). Chronic fascioliasis showed smaller liver sizes, firm consistency, and the presence of a corrugated capsule (Figure 4d). Black minute granules were also observed, indicative of hematoporphyrin pigment with a palpable gritty sensation in the bile ducts, and calcified bile duct wall. Mature liver flukes were occasionally observed within the lumens of thickened bile ducts (Figures 4b, c).

**Figure 5:**
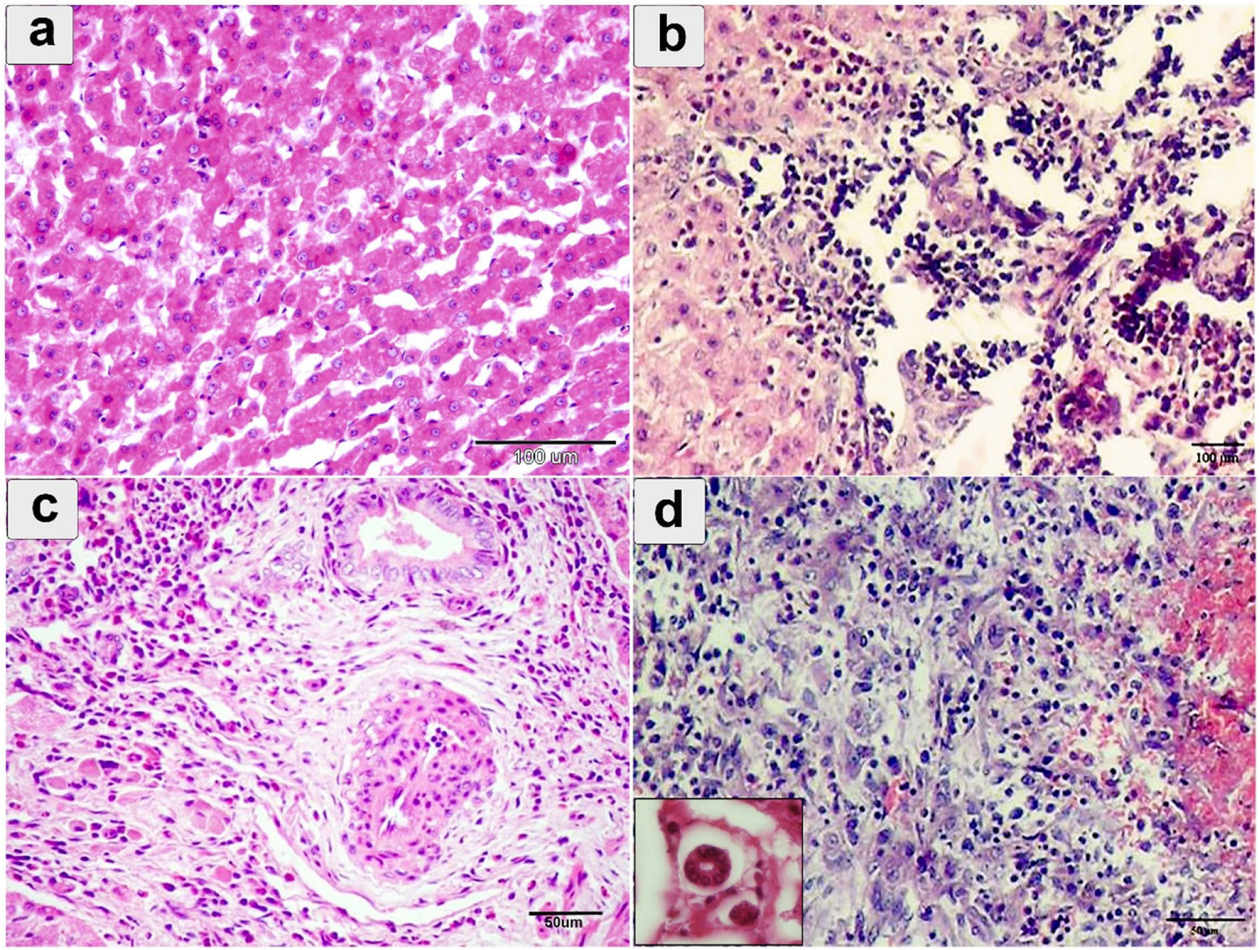
Histopathological examination of cattle-infected liver showing **(a)** coagulative necrosis of hepatic cells with destructed hepatocytes intermixed with eosinophilic infiltration; **(b)** hepatic sinusoids, fresh hemorrhage, Lymphohistiocytic infiltration, and cell necrosis; **(c)** Portal area showing endothelial hyperplasia of portal vessel, perivascular, and periductal fibrosis; **(d)** severe eosinophilic and lymphocytic cellular infiltration in the portal area. Giant cells were noted in small box. Staining: hematoxylin and Eosin.

Microscopically, the hepatocyte showed degeneration, coagulative necrosis, and subsequent destruction (Figure 5a). This involved the development of a migratory tract due to the parasite's traversal through hepatic tissues (Figure 5b). Severe dilation and congestion of the blood vessels including central veins, portal veins, and even small blood capillaries were observed. The portal area showed endothelial hyperplasia of the portal vessel, perivascular, and periductal fibrosis with forming new bile ducts (Figure 5c). Extensive infiltration of mononuclear cells, eosinophils, fibroblasts, and giant cells (Figure 5d).

**Figure 6:**
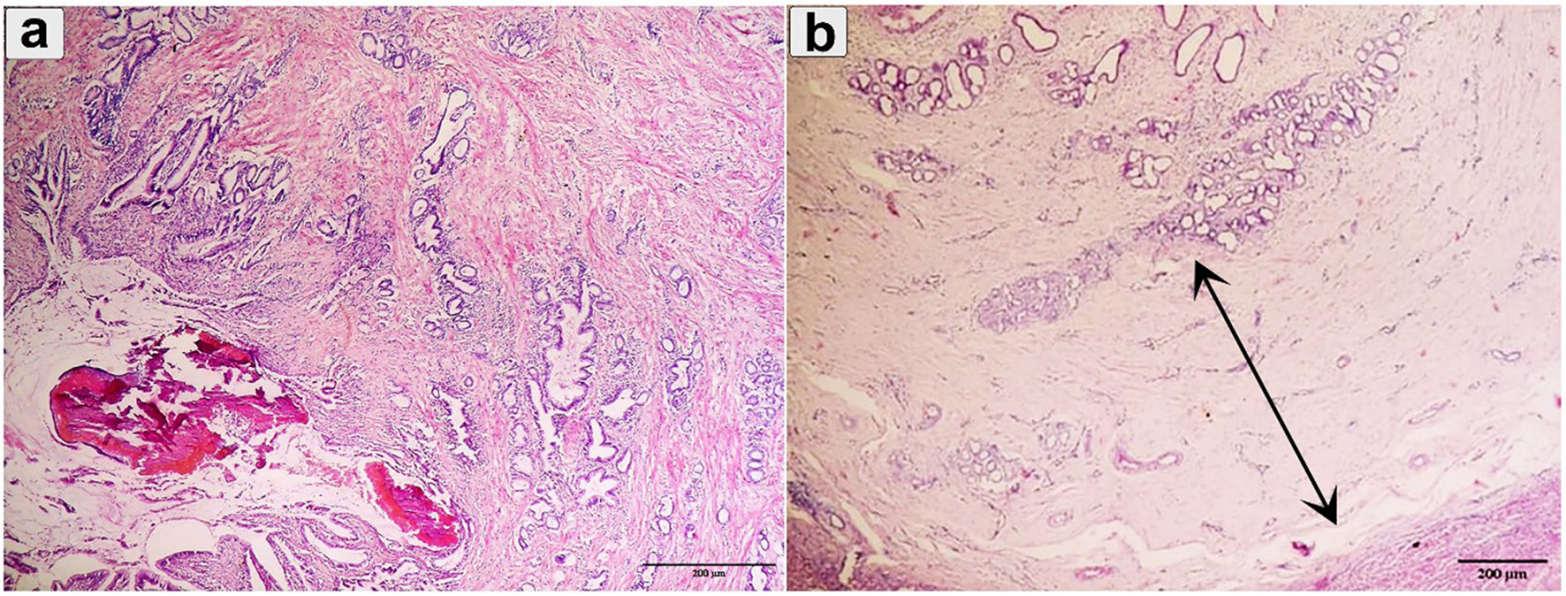
Histopathological examination of cattle-infected liver showing **(a)** Chronic cholangitis and bile duct hyperplasia was observed with multilobular cirrhosis and calcified parasite; **(b)** Bile duct hyperplasia with desquamation was observed (double arrow). Staining: hematoxylin and Eosin.

Chronic cholangitis, severe bridged fibrosis, periductal fibrosis, and dystrophic calcification, thickened calcified bile duct wall, and the development of multilobular cirrhosis were observed in chronic fasciolosis (Figures 6a, b).

#### Ectopic migration

**Figure 7:**
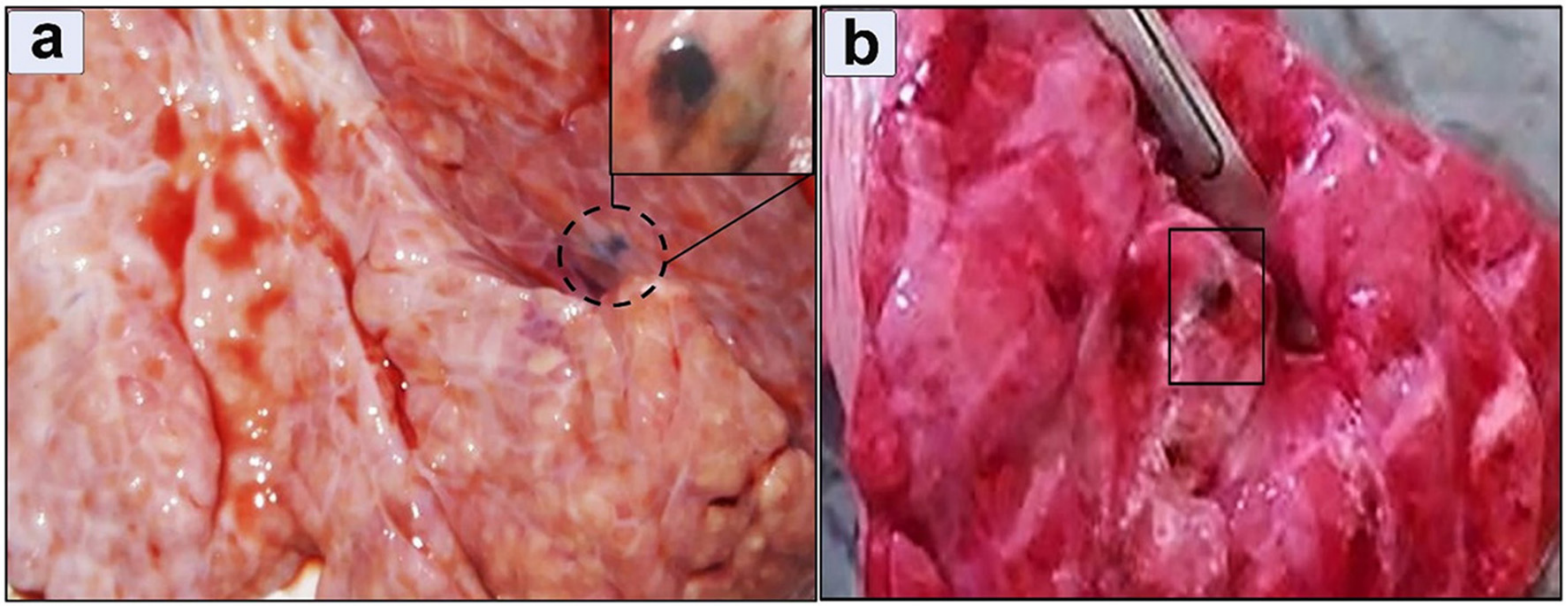
Gross appearance of a lung infected with *Fasciola hepatica* **(a)** Immature flukes encapsulated in the pulmonary parenchyma, **(b)** moist texture revealed after incision.

Ectopic migration in the lung was observed in 8 positive cases (3.5%) in which the pulmonary tissue showed a firm texture with a limited number of encapsulated immature flukes (Figure 7a). The encapsulation process was evident. The consolidated lobules had a moist texture. Notably, small airways within the tissue exhibited signs of leakage, characterized by purulent exudate (Figure 7b).

**Figure 8:**
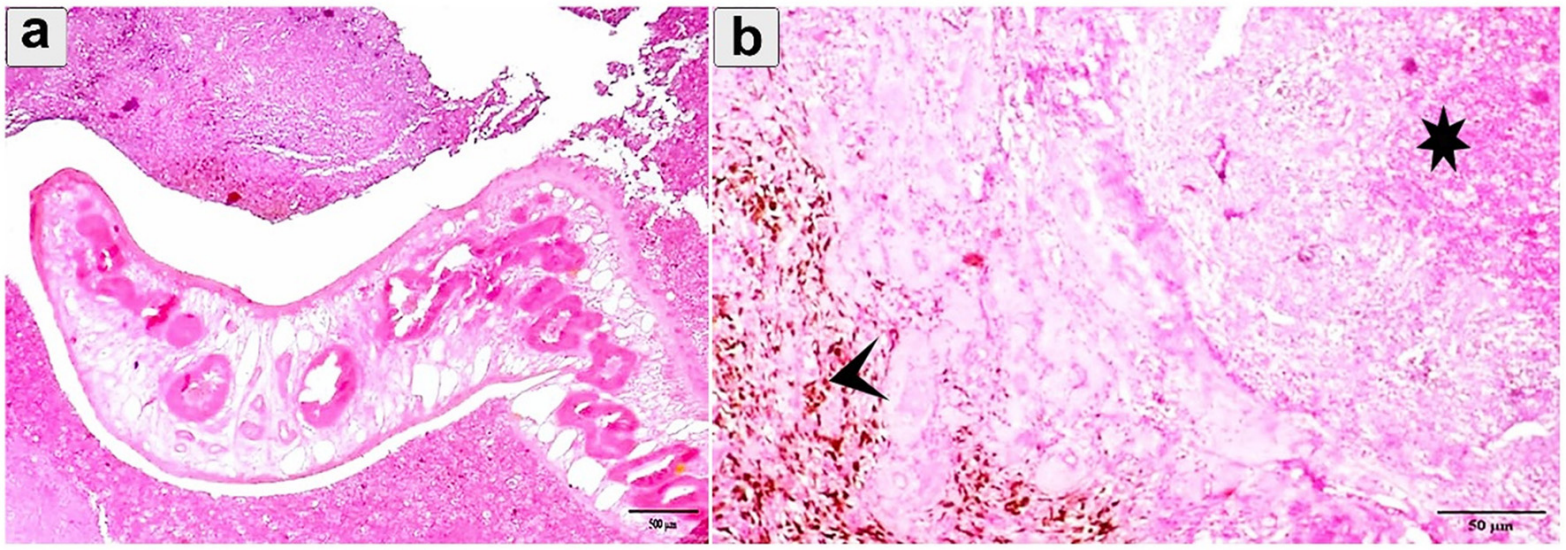
Histopathological examination of cattle-infected lungs showing **(a)** An immature fluke encapsulated by connective tissue with multiple necrosis and hemorrhage; **(b)** Eosinophilic infiltration and hemorrhage (asterisk) between alveoli. Prominent hemosiderosis was observed (arrowhead). Staining: hematoxylin and Eosin.

Histopathological examination revealed profound pathological alterations intricately linked to the parasite's presence. An immature fluke was observed within the lung tissue, encapsulated by connective tissue (Figure 8a). Concurrently, parasitic activity induced extensive necrosis, hemorrhage, and hemosiderosis in the alveoli (Figure 8b). In One case the flukes were not morphologically distinguishable and further molecular studies were needed.

#### Genetic characterization for Fasciola species in the pulmonary tissue

**Figure 9:**
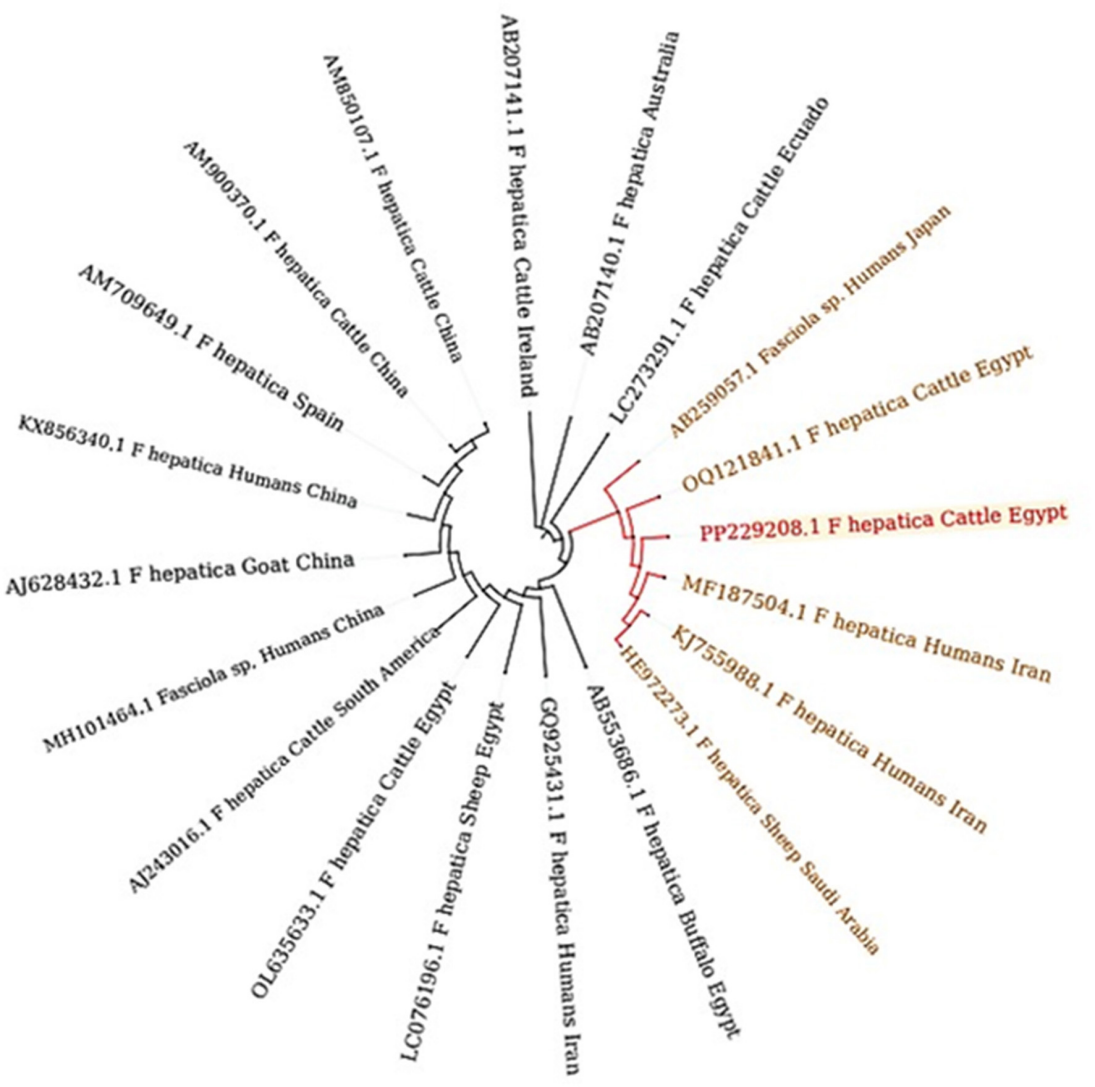
Phylogenetic tree constructed from ITS1 gene sequences, revealing distinct clades that signify discernible genetic lineages within *Fasciola hepatica*. The bootstrap confidence level inferred from 1,000 replicates is taken next to the branches.

The amplification of the *ITS1* gene from Fasciola DNA revealed a distinct band at 680 bp under UV light on a 1.5% agarose gel (Figure 9). Subsequent sequencing of the amplified gene and Blasting in NCBI showed a high identity of 98–100% with *Fasciola hepatica* (Figure 9), which was deposited in GenBank (accession number: PP229208).

#### Phylogenetic analysis

The phylogenetic relationship of the ITS1 nucleotide sequences from our isolate and 16 reference strains of *Fasciola* species, including *Fasciola hepatica, Fasciola gigantica*, and *Fasciola nyanzae*, with *Fascioloides magna* as the outgroup, is shown in Figure 9. *F. hepatica* clusters with our isolate (PP229208.1), supported by high bootstrap values (99 and 94%), highlighting their genetic similarity. In contrast, *F. gigantica* is clearly separated, with a bootstrap value of 91%, indicating divergence from *F. hepatica*. *F. nyanzae* forms a distinct clade, reflecting its unique evolutionary lineage, while *F. magna* is positioned on a separate branch, reinforcing its taxonomic distinction from the *Fasciola* species.

#### Genetic affinity of F. hepatica isolate

**Figure 10:**
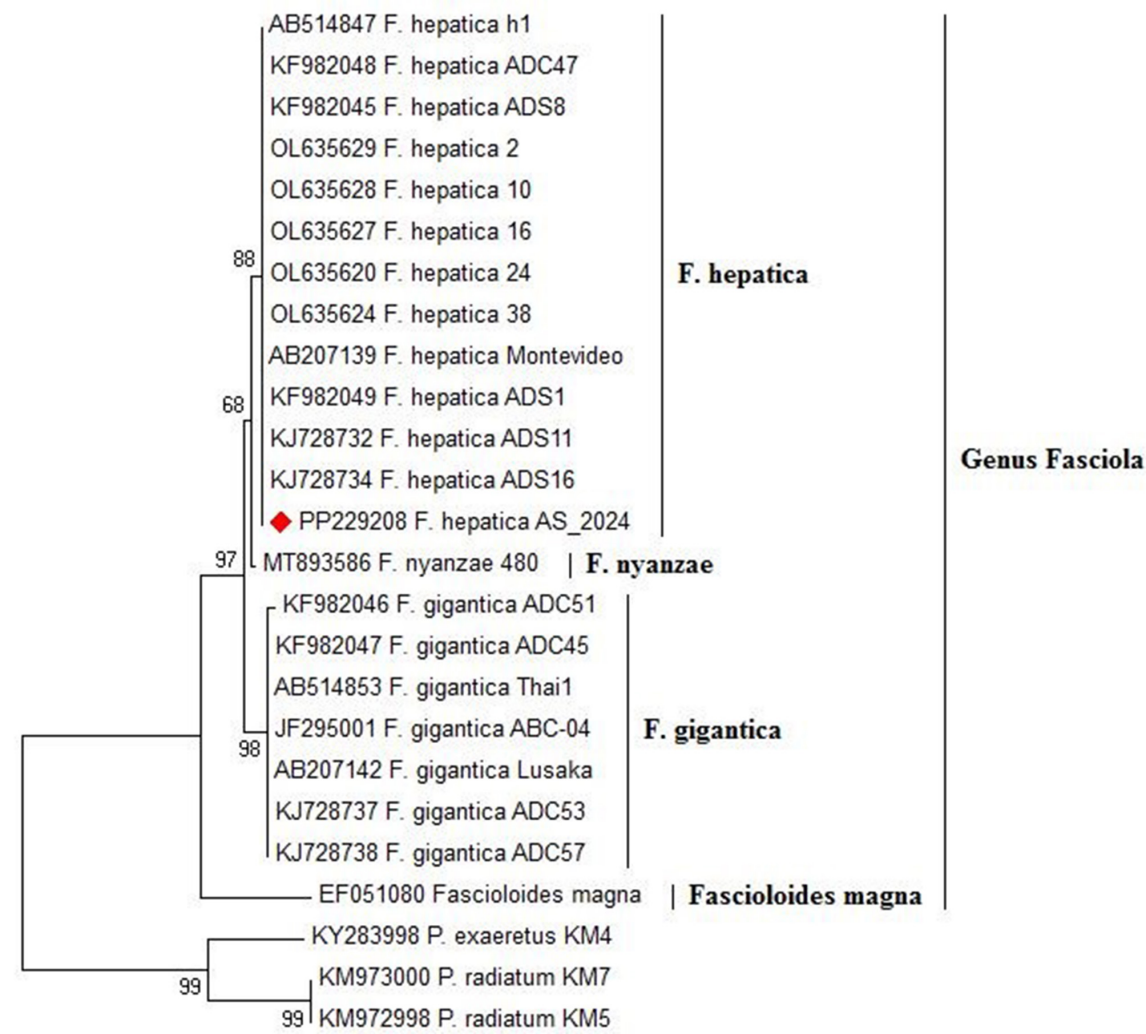
Phylogenetic relationships of *Fasciola* spp. based on the ITS-1 region, depicting genetic affiliations of *Fasciola hepatica* specimens from this study (PP229208.1) in comparison with representative isolates from GenBank across diverse hosts and geographic regions.

A comparative analysis between ~19 reference samples obtained from Egypt and other countries was conducted (Figure 10). There was a remarkable genetic similarity between the present *F. hepatica* isolate and the specimens previously isolated from cattle in Egypt, as well as from humans in Iran. This finding underscores the potential interconnectedness of *Fasciola* transmission between different host species and geographical regions.

## Discussion

The present study aimed to identify demographic, epidemiological, clinical manifestations, pathological, and genetic characteristics in both human and cattle populations diagnosed with fascioliasis in New Valley Governorate, Upper Egypt. In this area, factors such as water contamination with snails, poor management practices, and inadequate sanitary hygiene significantly contribute to the increase in fasciolosis infections among humans and animals. Although climatic conditions, temperature and humidity are important for the transmission and life cycle of *Fasciola* spp., with egg hatching inhibited below 10°C and optimal between 20 and 30°C, higher temperatures accelerate miracidia hatching and cercarial shedding in snails. Metacercarial viability decreases at elevated temperatures but is extended by higher humidity, while snail growth rates peak at 25 °C (35). The region's topography and climatic changes create conditions that further support the life cycle of *Fasciola*.

In the human population, our findings revealed that 58.3% (7/12) and 41.7% (5/12) were positively diagnosed through coprological analysis and radiological examination, respectively. Globally, Lan et al. (36) reported that the pooled human fasciolasis was 5%. Recent research demonstrated a high prevalence of fascioliasis infection, with ~20.4% of patients being affected, highlighting the significant public health concern posed by this parasite (37). Our findings align with a retrospective study conducted in Assiut, Egypt, which indicated a significant prevalence of the disease, recording ~261 cases between January 2018 and January 2020 (38). In another study in Upper Egypt (Manfalout, Assiut) on 67 patients infected with *Fasciola* spp. which indicate the endemicity of the infection within this region (39).

Additionally, recent research aimed at determining the global prevalence of *Fasciola* sp. in humans utilized a systematic review and meta-analysis of 4,422 articles, with 371 included in the final analysis. In the present study, analysis of infection distribution across different age groups indicated a notable concentration within the 31–42 age bracket, constituting 41.7% of cases, which corresponds to findings from a previous study by Ibrahim et al. (38), which reported that more than 40% of patients fell within the 21–40-years-old age. Regarding gender distribution, females appeared to be disproportionately affected by fascioliasis, comprising 66.7% of the infected individuals compared to 33.3% of males, consistent with recent previous reports by Hussieun et al. (40). The high incidence of infection in females can be influenced by behavioral factors and environmental exposure. Women typically handle cooking and vegetables, which places them in direct contact with potential sources of infection (encysted metacercaria). Additionally, pregnancy can elevate stress levels, further increasing their risk. Triclabendazole (TCBZ) emerged as the recommended drug for the treatment of acute and chronic human fascioliasis (41). Our findings showed that most patients received one or two doses of TCBZ, as recommended by the Centers for Disease Control and Prevention (42). Furthermore, a study by Branco et al. (43) found that treatment failure occurred in some cases of human fascioliasis even after patients received three doses. This observation may suggest a potential resistance against the anthelmintic drug, indicating variability in the efficacy of TCBZ treatment depending on the dosage regimen.

The overall prevalence of *Fasciola* infection among examined cattle was 23%. This finding aligns with various studies that have reported high prevalence rates of *Fasciola* infection in cattle across different regions. Diaz-Quevedo et al. (44) reported a high prevalence of cattle fascioliasis (90.13%) in Amazonas, Peru. Moreover, Lan et al. (36) showed the global pooled prevalence of bovine fasciolosis was 17%.

A noteworthy finding of our study is the variation in *Fasciola* infection prevalence across different age groups. Within the enrolled cattle, individuals aged > 3 years demonstrated the highest infection rates, with a prevalence of 30.36%, which aligns with Kusumarini et al. (45). This highlights the vulnerability of this age group to *Fasciola* infection, suggesting a need for targeted intervention strategies. In the present study, male animals often show higher infection rates than females, due to the high number of males that slaughtered at the abattoir house than females, this aligns with findings from Isah (46).

Regarding gender-based differences, our results showed a slightly higher prevalence of *Fasciola* infection in male animals 24.9% compared to females 20% in agreement with Mathewos et al. (12). However, gender may only have a minor influence on the risk of infection, with other factors likely playing a more substantial role in determining susceptibility. The analysis of seasonal variations in *Fasciola* infection revealed distinct patterns. The autumn season exhibited the highest infection rate of 28.7%, winter at 24.8%, followed by spring at 22.5% and summer at 18%. This observation is consistent with another study that demonstrated an increased risk of liver fluke infection in cattle slaughtered during August and November (47). The seasonal pattern in *Fasciola* infection may be attributed to environmental factors such as temperature and moisture, which influence the survival and availability of *Fasciola* spp. larvae in the environment (48).

Diagnosis of *Fasciola* infection involved a comprehensive approach utilizing three distinct methods: clinical manifestation, coprological analysis, and postmortem inspection. These diagnostic strategies yielded results consistent with previous reports, which successfully identified *Fasciola* in liver tissue through coprological and postmortem examination (10, 12). Morphologically, we have identified a distinct morphological feature in the parasite, including the total length (20 mm ± 5), oral sucker, ventral sucker, and genital pore with a slightly protruded cirrus which plays a vital role in reproduction and egg release. The spines in the anterior region of the parasite enhance its grip within the host's tissues. Additionally, the surface of the parasite's tegument, which is marked by micro ridges serves multiple functions, including increasing absorption of the surface area and potentially providing structural support or protection. These observations significantly enhance our understanding of *Fasciola* spp. anatomical characteristics and adaptations to parasitic life cycles. Genetic characterization of *Fasciola* species plays a crucial role in unraveling their evolutionary history, population genetics, and epidemiology. In this study, PCR amplification of the ITS1 gene from *Fasciola* DNA extracted from lung tissue produced distinct 680 bp bands. Sequencing of these bands revealed a high identity of 98–100% with *Fasciola hepatica*, these results harmonized with the recent findings in Egypt (8). Phylogenetic analysis further unveiled a close genetic affinity between *F. hepatica* and *F. gigantica* (2). Moreover, the study identified close sequence similarities to *F. nyanzae* and *F. magna*, indicating a direct evolution of *F. hepatica* and *F. gigantica* from *F. nyanzae* (49). The genetic similarity observed between our *F. hepatica* isolates and specimens previously isolated from cattle in Egypt and humans in Iran suggests the potential for interspecies transmission and zoonotic transmission of *F. hepatica* (8). The genetic resemblance between our isolate and specimens from both cattle and humans underscores the importance of one health approach in studying and managing fascioliasis (50). Furthermore, the genetic similarity across geographical regions, particularly between Egypt and Iran, hints at the potential dispersal of *F. hepatica* populations across borders (51). This could be facilitated by factors such as animal trade, migration, or natural dispersal mechanisms (52). Understanding the epidemiology of *F. hepatica* in different populations is essential for implementing effective control measures and preventing the spread of infection.

Microscopic analysis of liver specimens revealed a spectrum of fascioliasis infection, with 69.6% acute and 30.4% chronic manifestations. Acute cases showed enlarged, congested livers with signs of bleeding, while chronic cases presented smaller liver sizes and a corrugated capsule. These findings are consistent with Ashoor and Wakid (53) in Saudi Arabia, the presence of hemorrhagic migrating tracts emphasizes the destructive nature of migrating parasites on hepatocytes and erythrocytes. Additionally, chronic cholangitis and bile duct hyperplasia were prominent, often accompanied by the presence of dead and calcified parasites (9, 53). Furthermore, examination of lung tissue revealed the presence of firm tissue containing encapsulated immature flukes (3.5%), alongside areas of necrosis and hemorrhage, The same results were reported by Sotohy et al. (54).

Furthermore, alongside *Fasciola* infection, concurrent parasitic infections were reported, including *Paramphistomum* spp. (10.4%), *Moniezia* spp. (45.3%), *Avitellina* spp. (3.9%), and parasitic nodules linked to *Oesophagostomum* sp. infection (31.3%). These co-infections highlight the intricate nature of parasitic infestations in cattle, as previously noted in a study examining the simultaneous presence of both fascioliasis and trypanosomosis infections in cattle (55), indicating the coexistence of multiple parasitic species simultaneously.

The One Health perspective emphasizes the interconnectedness of human, animal, and environmental health. Controlling fasciolosis is vital for protecting both human and cattle health, as *Fasciola* spp. primarily affect the livestock. The disease imposes significant economic burdens on agriculture, reducing productivity and increasing veterinary costs. Infected cattle can act as reservoirs, facilitating transmission to humans, especially in shared water sources. Implementing control measures—such as regular livestock screening, improved sanitation, and community education—is essential. This integrated approach not only reduces disease incidence in humans but also enhances agricultural productivity, promoting healthier ecosystems and food security for all. Despite the retrospective studies including animal and human fasciolasis remains insufficient, which raises the findings of the present study. This study may have some limitations, including the sample size of the human population, and the determination of the specificity and sensitivity of diagnosis methods. Future study on the correlation between infection and heredity in human and animal populations is recommended.

## Conclusion

This study sheds light on the impact of *F. hepatica* infection on human and animal health, emphasizing its global prevalence as a neglected tropical disease with zoonotic potential. We have elucidated key insights into the prevalence, clinical manifestations, morphological analyses, histopathological examinations, and genetic diversity of *Fasciola* species. Genetic characterization provides valuable insights into its evolutionary history and population genetics. The close genetic affinity observed between *Fasciola* hepatica isolates from different host species and geographical regions highlights the need for integrated One Health approaches to disease control and prevention.

## Data Availability

The datasets presented in this study can be found in online repositories. The names of the repository/repositories and accession number(s) can be found in the article/supplementary material.

## References

[1] Dermauw V Muchai J Al Kappany Y Fajardo Castaneda AL Dorny P. Human fascioliasis in Africa: a systematic review. PLoS One. (2021) 16:e0261166. 10.1371/journal.pone.0261166 34882738 PMC8659297

[2] Flores-Velázquez LM Ruiz-Campillo MT Herrera-Torres G Martínez-Moreno Á Martínez-Moreno FJ Zafra R. Fasciolosis: pathogenesis, host-parasite interactions, and implication in vaccine development. Front Vet Sci. (2023) 10:1270064. 10.3389/fvets.2023.1270064 38149297 PMC10750376

[3] Cwiklinski K Donnelly S Drysdale O Jewhurst H Smith D Verissimo CDM. The cathepsin-like cysteine peptidases of trematodes of the genus *Fasciola*. Adv Parasitol. (2019) 104:113–64. 10.1016/bs.apar.2019.01.001 31030768

[4] Vázquez AA Alda P Lounnas M Sabourin E Alba A Pointier JP. Lymnaeid snails hosts of Fasciola hepatica and Fasciola gigantica (Trematoda: Digenea): a worldwide review. CABI Rev. (2018) 13:1–15. 10.1079/PAVSNNR201813062

[5] Beesley NJ Caminade C Charlier J Flynn RJ Hodgkinson JE Martinez-Moreno A. Fasciola and fasciolosis in ruminants in Europe: Identifying research needs. Transbound Emerg Dis. (2018) 65:199–216. 10.1111/tbed.12682 28984428 PMC6190748

[6] Abdel-Hakeem S Omar M. Ovine fascioliasis: environmental epidemiology and meta-analysis of the prevalence, agro-ecological and economic factors in five provinces of the Nile Delta region of Egypt. Damanhour J Vet Sci. (2020) 3:23–31. 10.21608/djvs.2020.67811

[7] Abdel-Hakeem SS Khalifa MM Mohammad WA. Parasitic contamination of two commonly consumed leafy vegetables in el-kharga oasis, upper egypt, and evaluation of hygiene practices among the vendors. J Egypt Soc Parasitol. (2021) 51:289–96. 10.21608/jesp.2021.193305

[8] Ahmad AA Ramadan HK-A Hassan WA Hakami MA Huseein EAM Mohamed SA-A. New perspectives for fascioliasis in Upper Egypt's new endemic region: sociodemographic characteristics and phylogenetic analysis of *Fasciola* in humans, animals, and lymnaeid vectors. PLOS Neglect Trop Dis. (2022) 16:e0011000. 10.1371/journal.pntd.0011000 36576925 PMC9797099

[9] Taibi A Aissi M Harhoura K Zenia S Zait H Hamrioui B. Evaluation of *Fasciola hepatica* infections in cattle in northeastern Algeria and the effects on both enzyme and hepatic damage, confirmed by scanning electron microscopy. Acta Parasitol. (2019) 64:112–28. 10.2478/s11686-018-00013-9 30689191

[10] Lalor R Cwiklinski K Calvani NED Dorey A Hamon S Corrales JL. Pathogenicity and virulence of the liver flukes *Fasciola hepatica* and *Fasciola gigantica* that cause the zoonosis Fasciolosis. Virulence. (2021) 12:2839–67. 10.1080/21505594.2021.1996520 34696693 PMC8632118

[11] Calvani NED Šlapeta J. *Fasciola* species introgression: just a fluke or something more? Trends Parasitol. (2021) 37:25–34. 10.1016/j.pt.2020.09.008 33097425 PMC7575431

[12] Mathewos M Endale H Kebamo M. Coprological and postmortem assessment and economic significance of bovine fasciolosis in cattle slaughtered at Tarcha Municipal Abattoir, Southern Ethiopia. Parasit Epidemiol Control. (2023) 22:e00316. 10.1016/j.parepi.2023.e00316 37521359 PMC10374966

[13] Garcia-Corredor D Alvarado M Pulido-Medellín M Muñoz M Cruz-Saavedra L Hernández C. Molecular characterization of *Fasciola hepatica* in endemic regions of Colombia. Front Vet Sci. (2023) 10:1171147. 10.3389/fvets.2023.1171147 37360412 PMC10288157

[14] Caravedo MA Cabada MM. Human fascioliasis: current epidemiological status and strategies for diagnosis, treatment, and control. Res Rep Trop Med. (2020) 26:149–58. 10.2147/RRTM.S237461 33273878 PMC7705270

[15] Drescher G. Vasconcelos TCBd, Belo VS, Pinto MMdG, Rosa JdO, Morello LG, et al. Serological diagnosis of *fasciolosis (Fasciola hepatica*) in humans, cattle, and sheep: a meta-analysis. Front Vet Sci. (2023) 10:1252454. 10.3389/fvets.2023.1252454 37736397 PMC10509555

[16] Sumruayphol S Siribat P Dujardin J-P Dujardin S Komalamisra C Thaenkham U. *Fasciola gigantica, F. hepatica* and Fasciola intermediate forms: geometric morphometrics and an artificial neural network to help morphological identification. PeerJ. (2020) 8:e8597. 10.7717/peerj.8597 32117632 PMC7034386

[17] Itagaki T Kikawa M Sakaguchi K Shimo J Terasaki K Shibahara T. Genetic characterization of parthenogenic *Fasciola* sp. in Japan on the basis of the sequences of ribosomal and mitochondrial DNA. Parasitology. (2005) 131:679–85. 10.1017/S0031182005008292 16255826

[18] Alsulami MN Mohamed K Wakid MH Abdel-Gaber R Timsah AG Al-Megrin WAI. Molecular Characterization of *Fasciola hepatica* in sheep based on DNA sequences of ribosomal ITS-1. Infect Drug Resist. (2023) 6661-71. 10.2147/IDR.S421206 37849790 PMC10578168

[19] Javanmard E Ohari Y Sadeghi A Cheraghipour K Aghdaei HA Mirjalali H. Multigene typing and phylogenetic analysis of Fasciola from endemic foci in Iran. Infect Genet Evol. (2020) 80:104202. 10.1016/j.meegid.2020.104202 31978563

[20] Rehman ZU Zahid O Rashid I Ali Q Akbar MH Oneeb M. Genetic diversity and multiplicity of infection in *Fasciola gigantica* isolates of Pakistani livestock. Parasitol Int. (2020) 76:102071. 10.1016/j.parint.2020.102071 32045674

[21] Hassan NA Diab MS Bayoumi AM Zidan SA Hadad GAEM. Epidemiological study on fascioliasis in animals and human in new valley governorate and evaluation of risk factors. New Valley Vet J. (2025) 5:42–9. 10.21608/nvvj.2024.322991.1052

[22] Elshraway NT Mahmoud WG. Prevalence of fascioliasis (liver flukes) infection in cattle and buffaloes slaughtered at the municipal abattoir of El-Kharga, Egypt. Vet World. (2017) 10:914. 10.14202/vetworld.2017.914-917 28919682 PMC5591478

[23] Du Sert NP Ahluwalia A Alam S Avey MT Baker M Browne WJ. et al. Reporting animal research: explanation and elaboration for the ARRIVE guidelines 20. PLoS Biol. (2020) 18:e3000411. 10.1371/journal.pbio.3000411 32663221 PMC7360025

[24] Kotsias G Lolis CJ Hatzianastassiou N Lionello P Bartzokas A. An objective definition of seasons for the Mediterranean region. Int J Climatol. (2021) 41:E1889–E905. 10.1002/joc.6819

[25] Abdel-Hakeem SS Fadladdin YA El-Sagheer AM Adel A. New host record, *Sclerophrys regularis* (Bufonidae), for *Rhabdias africanus* (Rhabdiasidae, Kuzmin, 2001) based on molecular and morphological evidence. Saudi J Biol Sci. (2022) 29:103366. 10.1016/j.sjbs.2022.103366 35860497 PMC9289862

[26] Gibbons LM Jones A Khalil LF. Manual for the 8th International Training Course on Identification of Helminth Parasites of Economic Importance. Wallingford: CAB International. (1996).

[27] Abdel-Hakeem SS Abdel-Samiee MA Abed GH. An insight into the potential parasitological effect of Schistosoma mansoni antigens in infected mice: prophylactic role of cercarial antigen. Microscop Microanaly. (2020) 26:708–16. 10.1017/S1431927620001695 32624059

[28] Abd-ELrahman SM Dyab AK. Mahmoud AE-s, Mohamed SM, Fouad AM, Gareh A, et al. Therapeutic effects of myrrh extract and myrrh-based silver nanoparticles on Trichinella spiralis-infected mice: parasitological, histopathological, and immunological (IFN-γ, IL-10, and MMP-9) investigations. Front Vet Sci. (2024) 11:1433964. 10.3389/fvets.2024.1433964 39421828 PMC11483346

[29] Dar Y Amer S Mercier A Courtioux B. Dreyfuss G. Molecular identification of Fasciola spp (*digenea: Fasciolidae*) in Egypt. Parasite. (2012) 19:177. 10.1051/parasite/2012192177 22550630 PMC3671433

[30] Abd-Elrahman SM Abdel-Rahman SM Bakir HY Othman RA Khedr AA Khalifa MM. Genetic relatedness and diversity of *Capillaria* species infecting bayad (*Bagrus bajad*) in upper Egypt. BMC Vet Res. (2024) 20:235. 10.1186/s12917-024-04076-x 38822316 PMC11141003

[31] Thompson JD Higgins DG Gibson TJ CLUSTAL W. improving the sensitivity of progressive multiple sequence alignment through sequence weighting, position-specific gap penalties and weight matrix choice. Nucl Acids Res. (1994) 22:4673–80. 10.1093/nar/22.22.4673 7984417 PMC308517

[32] Abd-Elrahman SM Dyab AK Kamel FA Khedr AA Khalifa MM Mohamed SM. Assessment of cattle tick infestation: molecular insights into *rhipicephalus annulatus* and the efficacy of garlic oil and nanoemulsion as acaricidal agents. Vet Parasitol. (2024) 329:110211. 10.1016/j.vetpar.2024.110211 38772086

[33] Pavlopoulos GA Soldatos TG Barbosa-Silva A Schneider R A. reference guide for tree analysis and visualization. BioData Min. (2010) 3:1–24. 10.1186/1756-0381-3-1 20175922 PMC2844399

[34] Mansour A Karar YF Hassan HA-S Mohamadain HS Abdel-Hakeem SS. Integrative supporting techniques for the taxonomy of Schistorchis carneus Lühe, 1906 (Digenea: Megaperidae) with perspective for the existence of species complexes. Zootaxa. (2025) 5569:299–327. 10.11646/zootaxa.5569.2.5 40173543

[35] Modabbernia G Meshgi B Kinsley AC. Climatic variations and *Fasciola*: a review of impacts across the parasite life cycle. Parasitol Res. (2024) 123:300. 10.1007/s00436-024-08319-6 39145846

[36] Lan Z Zhang X Xing J Zhang A Wang H Zhang X. Global prevalence of liver disease in human and domestic animals caused by Fasciola: a systematic review and meta-analysis. J Glob Health. (2024) 14:04223. 10.7189/jogh.14.04223 39297588 PMC11412093

[37] Rosas-Hostos Infantes LR Paredes Yataco GA Ortiz-Martínez Y Mayer T Terashima A. The global prevalence of human fascioliasis: a systematic review and meta-analysis. Therapeut Adv Infect Dis. (2023) 10:20499361231185413. 10.1177/20499361231185413 37434654 PMC10331341

[38] Ibrahim N Abdel Khalek EM Makhlouf NA Abdel-Gawad M Mekky M Ramadan HK-A. Clinical characteristics of human fascioliasis in Egypt. Sci Rep. (2023) 13:16254. 10.1038/s41598-023-42957-7 37758788 PMC10533839

[39] Ramadan HK-A Hassan WA Elossily NA Ahmad AA Mohamed AA Abd-Elkader AS. Evaluation of nitazoxanide treatment following triclabendazole failure in an outbreak of human fascioliasis in Upper Egypt. PLoS Neglect Trop Dis. (2019) 13:e0007779. 10.1371/journal.pntd.0007779 31553716 PMC6779272

[40] Hussieun SM Mohamed YM Bakir HY Othman RA. Abdel-rahman SM, Khalifa MM. Studies on sociodemography, clinical, laboratory, and treatment of fascioliasis patients in Assiut hospitals, Assiut Governorate, Egypt. J Egypt Soc Parasitol. (2022) 52:133–8. 10.21608/jesp.2022.235834

[41] Dondorp AM Dünser MW Schultz MJ. Emergency and Intensive Care Medicine in Resource-Poor Settings. Manson's Tropical Diseases, 4th Edn. Amsterdam: Elsevier (2023). p. 79-87. 10.1016/B978-0-7020-7959-7.00011-7

[42] Marcos L Maco V Terashima A. Triclabendazole for the treatment of human fascioliasis and the threat of treatment failures. Exp RevAnti-Infect Therapy. (2021) 19:817–23. 10.1080/14787210.2021.1858798 33267701

[43] Branco EA Ruas R Nuak J Sarmento A. Treatment failure after multiple courses of triclabendazole in a Portuguese patient with fascioliasis. BMJ Case Rep CP. (2020) 13:e232299. 10.1136/bcr-2019-232299 32193176 PMC7101001

[44] Diaz-Quevedo C Frias H Cahuana GM Tapia-Limonchi R Chenet SM Tejedo JR. High prevalence and risk factors of fascioliasis in cattle in Amazonas, Peru. Parasitol Int. (2021) 85:102428. 10.1016/j.parint.2021.102428 34329752

[45] Kusumarini S Permata F Widyaputri T Prasetyo D. Prevalence of fasciolosis emphasis on age, origin, body condition and post mortem by geographic information systems on sacrificial examination in Malang District–East Java. Journal of Physics: Conference Series. (2020): IOP Publishing. 10.1088/1742-6596/1430/1/012025

[46] Isah UM. Studies on the prevalence of fascioliasis among ruminant animals in northern Bauchi state, North-Eastern Nigeria. Parasit Epidemiol Control. (2019) 5:e00090. 10.1016/j.parepi.2019.e00090 30847412 PMC6393693

[47] Purwaningsih P Palulungan JA Tethool AN Noviyanti N Satrija F Murtini S. Seasonal dynamics of *Fasciola gigantica* transmission in Prafi district, Manokwari Regency, West Papua, Indonesia. Vet World. (2022) 15:2558. 10.14202/vetworld.2022.2558-2564 36590131 PMC9798046

[48] Hernández-Guzmán K Molina-Mendoza P Olivares-Pérez J Alcalá-Canto Y Olmedo-Juárez A Córdova-Izquierdo A. Prevalence and seasonal variation of *Fasciola hepatica* in slaughtered cattle: the role of climate and environmental factors in Mexico. J Helminthol. (2021) 95:e46. 10.1017/S0022149X21000444 34412711

[49] Bargues MD Halajian A Artigas P Luus-Powell WJ Valero MA Mas-Coma S. Paleobiogeographical origins of *Fasciola hepatica* and *F. gigantica* in light of new DNA sequence characteristics of F nyanzae from hippopotamus. Front Vet Sci. (2022) 9:990872. 10.3389/fvets.2022.990872 36157179 PMC9500510

[50] Mas-Coma S Valero MA Bargues MD. One Health for fascioliasis control in human endemic areas. Trends Parasitol. (2023) 39:650–67. 10.1016/j.pt.2023.05.009 37385922

[51] Ai L Chen M-X Alasaad S Elsheikha HM Li J Li H-L Lin R-Q. Genetic characterization, species differentiation and detection of *Fasciola* spp. by molecular approaches. Parasit Vector. (2011) 4:1–6. 10.1186/1756-3305-4-101 21658284 PMC3121690

[52] de Almeida TM Neto IR de Oliveira Brandão Y Molento MB. Geographic expansion of *Fasciola hepatica* (Linnaeus, 1758) due to changes in land use and cover in Brazil. Int J Parasitol. (2024) 54:201–12. 10.1016/j.ijpara.2023.12.003 38160740

[53] Ashoor SJ Wakid MH. Prevalence and hepatic histopathological findings of fascioliasis in sheep slaughtered in Jeddah, Saudi Arabia. Sci Rep. (2023) 13:6609. 10.1038/s41598-023-33927-0 37095133 PMC10126202

[54] Sotohy SA Hassan A Mahmoud W Khedr A. Prevalence and histopathological changes of bovine fascioliasis, with unusual migration to lung in New-Valley Governorate. Ass Vet Med J. (2019) 65:43–9. 10.21608/avmj.2019.168743

[55] Meharenet B Shitu D. Concurrent infection of fascioliasis and trypanosomosis and associated risk factors in local zebu breed Cattle of Western Ethiopia. Vet Med Res Rep. (2021) 12:15–22. 10.2147/VMRR.S285165 33564623 PMC7866923

